# A Narrative Review on the Pathophysiology of Preeclampsia

**DOI:** 10.3390/ijms25147569

**Published:** 2024-07-10

**Authors:** Johnatan Torres-Torres, Salvador Espino-y-Sosa, Raigam Martinez-Portilla, Hector Borboa-Olivares, Guadalupe Estrada-Gutierrez, Sandra Acevedo-Gallegos, Erika Ruiz-Ramirez, Martha Velasco-Espin, Pablo Cerda-Flores, Andrea Ramirez-Gonzalez, Lourdes Rojas-Zepeda

**Affiliations:** 1 Clinical Research Branch, Instituto Nacional de Perinatología Isidro Espinosa de los Reyes, Mexico City 11000, Mexico; raifet@hotmail.com (R.M.-P.);; 2 Obstetric and Gynecology Department, Hospital General de México Dr. Eduardo Liceaga, Mexico City 06720, Mexicopablocf9727@gmail.com (P.C.-F.);; 3 Maternal-Fetal Medicine Department, Instituto Materno Infantil del Estado de Mexico, Toluca 50170, Mexico

**Keywords:** preeclampsia, abnormal placentation, endothelial dysfunction, systemic inflammation, oxidative stress, pathophysiology

## Abstract

Preeclampsia (PE) is a multifactorial pregnancy disorder characterized by hypertension and proteinuria, posing significant risks to both maternal and fetal health. Despite extensive research, its complex pathophysiology remains incompletely understood. This narrative review aims to elucidate the intricate mechanisms contributing to PE, focusing on abnormal placentation, maternal systemic response, oxidative stress, inflammation, and genetic and epigenetic factors. This review synthesizes findings from recent studies, clinical trials, and meta-analyses, highlighting key molecular and cellular pathways involved in PE. The review integrates data on oxidative stress biomarkers, angiogenic factors, immune interactions, and mitochondrial dysfunction. PE is initiated by poor placentation due to inadequate trophoblast invasion and improper spiral artery remodeling, leading to placental hypoxia. This triggers the release of anti-angiogenic factors such as soluble fms-like tyrosine kinase-1 (sFlt-1) and soluble endoglin (sEng), causing widespread endothelial dysfunction and systemic inflammation. Oxidative stress, mitochondrial abnormalities, and immune dysregulation further exacerbate the condition. Genetic and epigenetic modifications, including polymorphisms in the Fms-like tyrosine kinase 1 (FLT1) gene and altered microRNA (miRNA) expression, play critical roles. Emerging therapeutic strategies targeting oxidative stress, inflammation, angiogenesis, and specific molecular pathways like the heme oxygenase-1/carbon monoxide (HO-1/CO) and cystathionine gamma-lyase/hydrogen sulfide (CSE/H2S) pathways show promise in mitigating preeclampsia’s effects. PE is a complex disorder with multifactorial origins involving abnormal placentation, endothelial dysfunction, systemic inflammation, and oxidative stress. Despite advances in understanding its pathophysiology, effective prevention and treatment strategies remain limited. Continued research is essential to develop targeted therapies that can improve outcomes for both mothers and their babies.

## 1. Introduction

Preeclampsia (PE) is a multifaceted syndrome that complicates approximately 3–5% of all pregnancies [1,2]. It is primarily identified by the onset of hypertension after the 20th week of gestation and is frequently accompanied by dysfunction in multiple organs, including the kidneys, liver, blood, brain, and placenta [3,4]. The clinical presentation of PE can vary significantly, ranging from mild to severe, with severe cases leading to life-threatening complications for both the mother and the fetus [4]. Women who have experienced PE are at an increased risk for chronic conditions such as chronic hypertension, cardiovascular disease, stroke, metabolic syndrome, cognitive impairment, and end-stage renal disease [5,6,7,8]. Moreover, PE poses significant risks to the fetus and the newborn. Infants born to mothers with PE are at an elevated risk for both immediate and long-term health issues. In the short term, these infants may suffer from complications related to preterm birth and intrauterine growth restriction [9]. In the long term, they are more susceptible to neurodevelopmental impairments, diabetes mellitus, coronary heart disease, and hypertension [10,11,12].

Risk factors for PE include a history of PE in previous pregnancies, chronic hypertension, preexisting diabetes, kidney disease, autoimmune disorders, obesity, advanced maternal age, multiple pregnancies, and certain genetic predispositions [2,13,14]. Additionally, first-time pregnancies and assisted reproductive technologies are associated with a higher risk of developing PE [2,15,16].

Despite extensive research, the precise mechanisms that lead to the development of PE remain largely unclear. It is widely recognized that the condition originates from abnormal placentation early in pregnancy, which subsequently leads to widespread endothelial dysfunction. This dysfunction results in the clinical manifestations of the disease, including hypertension and organ damage [2,14]. PE is an almost exclusively human condition, presenting significant challenges in the use of experimental models for its study. This exclusivity can be attributed to the unique characteristics of human placentation, including the deep invasion of trophoblasts into maternal spiral arteries and the complex immunological regulation at the maternal–fetal interface [17]. These peculiarities make it difficult to accurately reproduce the disease in animal models, limiting our understanding and the development of effective treatments.

This narrative review aims to synthesize recent findings and theories to provide a comprehensive overview of the pathophysiological processes involved in PE using recent evidence.

## 2. Results

### 2.1. The Etiology of Preeclampsia

**Figure 1:**
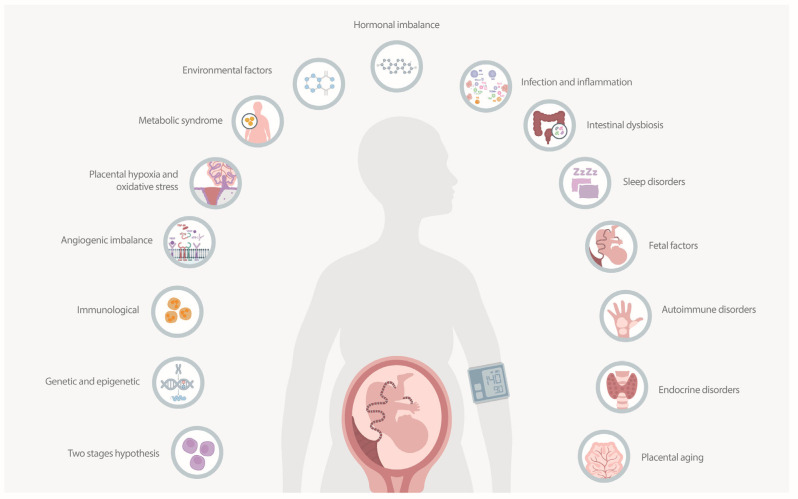
Hypotheses on the etiology of preeclampsia. The two-stage hypothesis suggests the condition develops from impaired placentation followed by a maternal systemic response. The genetic and epigenetic hypothesis focuses on genetic predispositions and epigenetic changes. The immunological hypothesis proposes abnormal maternal immune adaptation. The angiogenic imbalance hypothesis highlights the imbalance between pro-angiogenic and anti-angiogenic factors. The placental hypoxia and oxidative stress hypothesis emphasizes oxidative stress from placental hypoxia. Other contributing factors include metabolic syndrome, environmental agents, hormonal imbalances, maternal infections, intestinal dysbiosis, sleep disorders, fetal conditions, autoimmune disorders, endocrine disorders, and placental aging.

PE is a multifaceted condition with various contributing factors. Understanding its etiology is essential for developing effective preventive and therapeutic strategies [16]. Several hypotheses have been proposed to explain the development of PE, each highlighting different aspects of its pathogenesis. These hypotheses are summarized in Figure 1. The two-stage hypothesis suggests that PE develops in two stages: impaired placentation due to inadequate trophoblast invasion, leading to poor spiral artery remodeling and placental hypoxia, followed by a maternal systemic response involving the release of anti-angiogenic factors (soluble fms-like tyrosine kinase-1 (sFlt-1) and soluble endoglin (sEng)), causing widespread endothelial dysfunction and systemic inflammation [18,19]. The genetic and epigenetic hypothesis focuses on genetic predispositions and epigenetic changes, suggesting that genetic variants in both the mother and fetus affect placental development, while epigenetic modifications (DNA methylation, histone modification) influence gene expression related to immune response and angiogenesis [20,21]. The immunological hypothesis argues that abnormal maternal immune adaptation to the fetus triggers PE. The maternal immune system fails to adequately tolerate fetal antigens, leading to an imbalance in immune cells (natural killer (NK) cells and regulatory T cells (Tregs)) and resulting in inflammatory responses and inadequate spiral artery remodeling [22]. The angiogenic imbalance hypothesis highlights the disruption of balance between pro-angiogenic factors (vascular endothelial growth factor (VEGF), placental growth factor (PlGF)), and anti-angiogenic factors (sFlt-1, sEng), impairing blood vessel formation and function, which results in endothelial dysfunction and reduced placental perfusion [23]. The placental hypoxia and oxidative stress hypothesis suggests that oxidative stress due to placental hypoxia contributes to PE. Placental hypoxia increases the production of reactive oxygen species (ROS), which damage placental and maternal endothelial cells, impairing their function [24]. The metabolic syndrome hypothesis links preeclampsia to metabolic syndrome and associated conditions, including obesity, insulin resistance, and hypertension. Metabolic disturbances lead to systemic inflammation and endothelial dysfunction, contributing to PE development [25]. Additionally, PE is likely caused by a combination of factors. The environmental factors hypothesis emphasizes the impact of endocrine disruptors such as BPA and phthalates on hormone signaling pathways, inducing oxidative stress and inflammation affecting placental and vascular function. The hormonal imbalance hypothesis discusses the roles of estrogens and androgens in regulating angiogenic factors, endothelial function, and immune responses, with disruptions leading to oxidative stress, inflammation, and endothelial dysfunction. The infection and inflammation hypothesis suggests maternal infections and inflammation contribute to PE by inducing systemic inflammatory responses, increasing cytokine production, and causing placental inflammation from conditions such as periodontal disease, urinary tract infections, and COVID-19. The intestinal dysbiosis hypothesis proposes that maternal intestinal dysbiosis affects pregnancy outcomes by altering gut microbiota, increasing intestinal permeability, and modulating the immune system, leading to systemic inflammation and contributing to PE. The sleep disorders hypothesis links sleep disorders during pregnancy, such as sleep apnea and altered sleep patterns, to increased PE risk through increased sympathetic activity and inflammation. The fetal factors hypothesis explores how fetal conditions like hydrops fetalis, viral infections, trisomy 13, and complications from multiple gestations can lead to placental and systemic effects contributing to PE. The autoimmune disorders hypothesis examines how autoimmune conditions with chronic immune activation, autoantibody production, and systemic inflammation increase the risk of PE. The endocrine disorders hypothesis investigates how endocrine disorders like hyperparathyroidism, Cushing’s syndrome, and hyperaldosteronism cause hormonal imbalances, leading to altered vascular reactivity, increased blood pressure, and electrolyte disturbances. Finally, the placental aging hypothesis attributes PE to placental aging, characterized by telomere shortening, cellular senescence, oxidative stress, and impaired placental function, increasing PE risk [16,18,26].

Pathophysiology in the context of PE involves bodily changes and processes such as endothelial dysfunction, oxidative stress, and systemic inflammation that explain how the disease develops and progresses. This review emphasizes the framework of abnormal placentation and the subsequent maternal systemic response, highlighting the multifactorial nature of preeclampsia’s etiology. These stages encompass various molecular, genetic, and immunological factors that contribute to the disease’s progression and clinical manifestations (Table 1).

### 2.2. Abnormal Placentation

**Figure 2:**
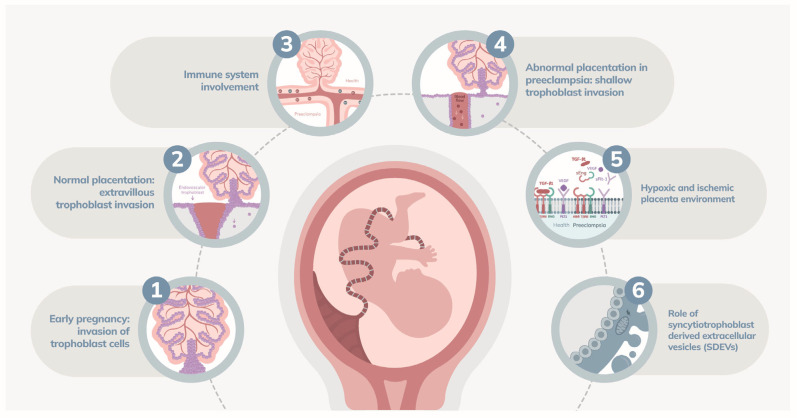
Mechanisms of abnormal placentation in preeclampsia. (**1**) Early in pregnancy, trophoblast cells invade the maternal decidua to establish a connection between maternal blood supply and the developing fetus. (**2**) In a healthy pregnancy, extravillous trophoblasts invade the maternal spiral arteries, transforming these high-resistance vessels into low-resistance channels, ensuring adequate blood flow to the placenta and fetus. (**3**) A balanced immune response is vital. Dysfunctional interactions between immune cells and trophoblasts in preeclampsia lead to inadequate spiral artery remodeling. (**4**) In preeclampsia, the invasion of trophoblasts is shallow, failing to reach the deeper segments of the spiral arteries. This leads to increased resistance and reduced blood flow to the placenta. (**5**) The inadequate remodeling creates a hypoxic and ischemic environment within the placenta, stimulating the release of pro-inflammatory and anti-angiogenic factors, leading to systemic endothelial dysfunction. (**6**) In preeclampsia, excess SDEVs carry bioactive molecules that induce inflammation, endothelial dysfunction, and coagulation abnormalities, exacerbating systemic endothelial dysfunction.

Abnormal placentation begins early in pregnancy and is characterized by insufficient invasion of trophoblast cells into the maternal decidua [29]. Normally, extravillous trophoblasts invade the maternal spiral arteries, transforming them into low-resistance channels to ensure adequate blood flow for fetal nutrition and growth [28]. In PE, this invasion is shallow, and the arteries retain their muscular and elastic properties, leading to increased resistance and reduced placental blood flow [27]. This results in a hypoxic and ischemic environment within the placenta, stimulating the release of pro-inflammatory and anti-angiogenic factors that contribute to systemic endothelial dysfunction [16] (Figure 2).

#### 2.2.1. Immune Response

In normal conditions, a balance between pro-inflammatory and anti-inflammatory responses supports trophoblast invasion and placental development [35]. Uterine NK cells, macrophages, and Tregs are crucial in facilitating this process [36]. Uterine NK cells, which are abundant in the decidua, interact with trophoblasts to promote their invasion and the remodeling of spiral arteries [37]. These interactions are mediated by receptors and ligands such as KIR (killer immunoglobulin-like receptors) on NK cells and HLA-C and HLA-G on trophoblasts [37,38].

In PE, these immune interactions are often dysfunctional. Uterine NK cells may fail to recognize and support trophoblast invasion effectively, leading to inadequate spiral artery remodeling. Additionally, an imbalance in cytokine production, with elevated levels of pro-inflammatory cytokines such as TNF-α and IL-6 and reduced levels of anti-inflammatory cytokines, contributes to endothelial dysfunction and impaired placentation [35,36,37,38]. The pro-inflammatory environment not only affects trophoblast invasion but also promotes oxidative stress, further damaging the placental tissue.

#### 2.2.2. Glycosylation and the Expression of Galectins

Glycosylation, a crucial post-translational modification, significantly influences protein function and cell signaling, both vital for placental development. In PE, aberrant glycosylation patterns disrupt cell–cell and cell–matrix interactions, leading to impaired trophoblast invasion, poor vascular remodeling, and heightened inflammation. This contributes to placental insufficiency and increased PE risk. For example, reduced expression of Sda-capped N-glycans hinders critical processes such as trophoblast invasion and spiral artery remodeling [39].

Galectins, a family of glycan-binding proteins, modulate various cellular processes essential during pregnancy. Galectin-1 (Gal-1) is key for maintaining immune tolerance, promoting angiogenesis, and facilitating trophoblast invasion. Deficient Gal-1 levels in early pregnancy are linked to PE development by impairing necessary immune and vascular adaptations. Gal-1-deficient mice exhibit PE-like symptoms, including hypertension and fetal growth restriction [40]. Galectin-3 (Gal-3) regulates immune responses and trophoblast functions. Dysregulation of Gal-3 in the placenta is associated with adverse pregnancy outcomes, including PE. Gal-3’s expression in trophoblasts and maternal decidual cells supports cell–matrix interactions crucial for placentation [41].

The interplay between glycosylation and galectins significantly impacts the behavior of trophoblasts and immune cells at the maternal–fetal interface. Abnormal glycosylation disrupts galectin-mediated signaling pathways, leading to endothelial dysfunction and increased inflammation, as seen in PE. Enhanced expression of specific glycosyltransferases in the placenta modifies glycan structures, affecting galectin binding and function, thus contributing to PE’s pathophysiology [39,41]. Understanding these mechanisms provides insights into potential therapeutic targets to improve placental function and maternal–fetal outcomes.

#### 2.2.3. Genetic Factors

The genetic expression of the non-classical major histocompatibility complex (MHC) class I molecule, HLA-G, by extravillous trophoblasts, is crucial to modulating immune tolerance to ensure adequate trophoblast invasion. Reduced expression of HLA-G results in inadequate immune response that impedes proper trophoblastic invasion [36].

#### 2.2.4. Apoptosis

Apoptosis regulates the turnover of trophoblast cells and ensures the proper development and function of the placenta [42]. However, in PE, there is excessive activation of apoptotic pathways in trophoblasts. Both extrinsic and intrinsic pathways of apoptosis are implicated, involving factors such as FAS and FAS ligand (FASL) interactions, as well as mitochondrial stress leading to the release of cytochrome C and activation of caspases [36,43]. This heightened apoptosis disrupts trophoblast invasion and spiral artery remodeling, contributing to placental insufficiency and hypoxia.

#### 2.2.5. Dyslipidemia

Pregnant women with PE often exhibit elevated levels of triglycerides, low-density lipoprotein cholesterol (LDL-C), and altered lipid metabolism. These abnormal lipid concentrations contribute to endothelial dysfunction and inflammation, further impairing placental development and function. Lipid-laden macrophages, or foam cells, can accumulate in the placenta [44,45,46]. This accumulation mimics atherosclerotic lesions seen in cardiovascular diseases, leading to a condition known as acute atherosis [47].

Acute atherosis in the placenta is characterized by the presence of these foam cells, fibrinoid necrosis of the vessel walls, and perivascular lymphocytic infiltration. These pathological changes result in a thickening and narrowing of the spiral arteries, reducing blood flow to the placenta. The reduced perfusion leads to placental hypoxia and ischemia, which exacerbates oxidative stress and inflammatory responses within the placental tissue. This vascular pathology not only compromises the placental function but also contributes to systemic endothelial dysfunction, perpetuating the cycle of hypertension and organ damage seen in PE [47,48].

#### 2.2.6. Role of Ion Channels in Trophoblast Invasion and Vascular Remodeling

Ion channels, including calcium, potassium, and sodium channels, are crucial for trophoblast invasion into the maternal decidua and the remodeling of maternal spiral arteries. Proper channel function ensures adequate blood flow to the placenta and the developing fetus. Calcium channels regulate intracellular calcium levels, essential for trophoblast motility and invasion, and their dysfunction can impair these processes, leading to inadequate placental development [49].

Potassium channels influence membrane potential and vessel contractility. Their activation leads to cell membrane hyperpolarization, closure of voltage-gated Ca^2+^ channels (VGCCs), reduced intracellular Ca^2+^, and vasodilation. Conversely, their closure causes depolarization, opening of VGCCs, increased intracellular Ca^2+^, and vasoconstriction. The human umbilical artery (HUA) contains various K^+^ channels, including voltage-dependent K^+^ (Kv) channels, large-conductance Ca^2+^-activated K^+^ (BKCa) channels, small-conductance Ca^2+^-activated K^+^ (SKCa) channels, inward rectifier K^+^ (Kir) channels, ATP-sensitive K^+^ (KATP) channels, and two-pore domain K^+^ (K2P) channels. These channels regulate vascular responses, maintaining tone and reactivity. In PE, abnormal regulation of K^+^ channels contributes to hypertension and endothelial dysfunction. Kv channels are essential for maintaining basal tone, and their reduced activity can lead to vasoconstriction. BKCa channels mediate hyperpolarization in response to elevated intracellular Ca^2+^, and their disruption leads to increased vascular resistance. Kir channels regulate membrane potential by allowing K^+^ influx at hyperpolarized potentials, and their dysfunction alters vascular reactivity. KATP channels link cellular metabolism to membrane excitability, and their compromised function impairs vascular relaxation [50].

#### 2.2.7. Syncytiotrophoblast-Derived Extracellular Vesicles

Syncytiotrophoblast-derived extracellular vesicles (SDEVs) are released from the syncytiotrophoblast layer of the placenta, carry a variety of bioactive molecules, including proteins, lipids, and RNA [51]. In PE, placental SDEVs can induce inflammation, endothelial dysfunction, and coagulation abnormalities in the mother [52]. SDEVs from preeclamptic placentas are particularly enriched with pro-inflammatory cytokines, anti-angiogenic factors like sFlt-1, and procoagulant molecules such as tissue factor and phosphatidylserine [51,53,54]. These vesicles exacerbate the maternal inflammatory response, contributing to the systemic endothelial dysfunction observed in PE [55].

### 2.3. Maternal Systemic Response

A critical aspect of PE is its close relationship with maternal cardiovascular health, both before and after pregnancy. PE and cardiovascular diseases share genetic and non-genetic risk factors, and epidemiological studies suggest that some cardiovascular risk factors also increase the risk of developing PE [56]. Furthermore, women with PE have been observed to have poorer long-term cardiovascular outcomes, indicating that the cardiovascular system may play a fundamental role in the pathogenesis of PE [57]. Normal maternal cardiovascular changes during pregnancy include increased cardiac output, expanded blood volume, and reduced systemic vascular resistance and blood pressure. In PE, conditions such as chronic hypertension or increased afterload can lead to pathological hypertrophy, characterized by concentric remodeling of the left ventricle. These hemodynamic alterations and cardiac dysfunction are observed both before and at the clinical onset of PE, underscoring the importance of the cardiovascular system in the pathogenesis of the disease [56,57]. Thus, the interplay between placental factors and maternal cardiovascular health is central to understanding the systemic response observed in PE.

**Figure 3:**
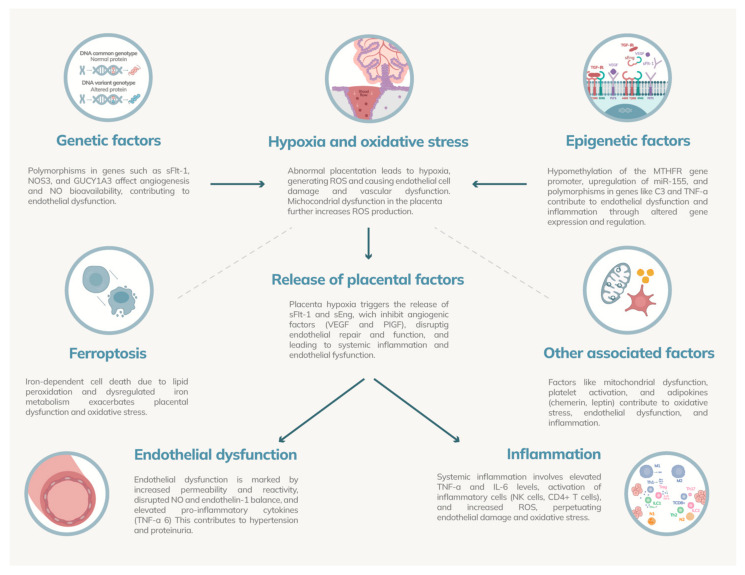
Maternal systemic response in preeclampsia. Genetic factors include polymorphisms in genes such as soluble fms-like tyrosine kinase-1 (sFlt-1), nitric oxide synthase 3 (NOS3), and guanylate cyclase 1 soluble subunit alpha 3 (GUCY1A3), which affect angiogenesis and nitric oxide bioavailability, contributing to endothelial dysfunction. Hypoxia and oxidative stress result from abnormal placentation, leading to reactive oxygen species production and endothelial damage. Epigenetic factors involve hypomethylation of the methylenetetrahydrofolate reductase (MTHFR) gene promoter and polymorphisms in genes like complement component 3 (C3) and tumor necrosis factor-alpha (TNF-α), contributing to inflammation. Placental hypoxia triggers the release of anti-angiogenic factors such as soluble endoglin (sEng) and sFlt-1, disrupting endothelial repair. Ferroptosis exacerbates oxidative stress through iron-dependent cell death. Endothelial dysfunction is marked by increased permeability and inflammatory cytokines. Additional factors include mitochondrial dysfunction, platelet activation, adipokines, and systemic inflammation involving elevated cytokines like interleukin-6 (IL-6), TNF-α, and reactive oxygen species (ROS), perpetuating endothelial damage and oxidative stress.

The second stage of PE is marked by a robust maternal systemic response to placental dysfunction. This response is primarily driven by the release of various placental factors into the maternal circulation, which subsequently induce widespread endothelial dysfunction and systemic inflammation [58] (Figure 3). Central to this pathophysiological process are sFlt-1 and sEng, among other factors, as detailed in Table 2, which summarizes the mechanisms by which these and other factors contribute to the condition [59].

#### 2.3.1. Pro-Angiogenic and Anti-Angiogenic Factors

Placental hypoxia, resulting from inadequate trophoblast invasion and poor spiral artery remodeling, triggers the overproduction of sFlt-1 and sEng [59]. sFlt-1 acts as a decoy receptor for VEGF and PlGF, binding and neutralizing these pro-angiogenic molecules. This inhibition of VEGF and PlGF disrupts angiogenesis and endothelial repair, leading to endothelial dysfunction. sEng, on the other hand, interferes with transforming growth factor-beta (TGF-β) signaling, further exacerbating endothelial damage [30]. This angiogenic disbalance is the key to endothelial dysfunction resulting in a reduced production of nitric oxide (NO) and clinical manifestations of PE such as proteinuria and hypertension [30,58]. Additionally, recent research highlights the role of CD93, a protein crucial for endothelial homeostasis and angiogenesis, in the pathophysiology of PE. Lower serum levels of CD93 in early pregnancy are associated with a higher risk of developing PE, indicating that defective angiogenesis and vascular dysfunction are key components in the disease’s progression [84].

#### 2.3.2. Endothelial Dysfunction

Endothelial dysfunction results in increased vascular permeability and heightened vascular reactivity [60]. A disbalance between vasodilators like NO and vasoconstrictors like endothelin-1 leads to vasoconstriction and increased blood pressure [85]. The decreased bioavailability of NO, due to the scavenging by ROS such as superoxide, further contributes to vasoconstriction and hypertension [58,59,86]. Additionally, the presence of sFlt-1 and sEng in the maternal circulation promotes a pro-inflammatory state, characterized by elevated levels of cytokines like tumor necrosis factor-alpha (TNF-α) and interleukin-6 (IL-6) [59].

#### 2.3.3. Oxidative Stress

The hypoxic placenta generates excess ROS, such as superoxide anion (O^2−^) and hydrogen peroxide (H_2_O_2_), primarily through the activities of NADPH oxidase and xanthine oxidase (XO) [65]. XO plays a crucial role in the oxidative stress associated with preeclampsia, a serious hypertensive disorder of pregnancy. XO is an enzyme involved in purine metabolism, catalyzing the conversion of hypoxanthine to xanthine and subsequently to uric acid. During these reactions, XO produces ROS such as superoxide and hydrogen peroxide, which contribute to oxidative stress. In PE, the activity of XO is heightened due to intermittent placental perfusion and hypoxia-reoxygenation injury, leading to an increased production of ROS. This oxidative stress damages endothelial cells, exacerbating endothelial dysfunction. This endothelial damage results in impaired vascular relaxation and contributes to the systemic inflammatory response [61,87]. Elevated levels of serum uric acid, a byproduct of XO activity, are commonly observed in PE. This hyperuricemia, particularly when adjusted for serum creatinine (SUA/sCr), is a significant marker of oxidative stress and vascular dysfunction [87]. Increased XO activity and the resulting oxidative stress are directly linked to the severity of PE and its complications, including hypertension and organ damage.

Mitochondrial dysfunction in the placenta also contributes to an overproduction of ROS [62]. These ROS damage cellular components, including lipids, proteins, and DNA, leading to endothelial cell apoptosis and furthering endothelial dysfunction [63]. The formation of peroxynitrite (ONOO−) from the reaction of NO with superoxide exacerbates oxidative damage and inflammation [65,88] as well as endothelial activation and injury [59]. Studies have shown that oxidative stress in PE is linked to increased mitochondrial ROS production and mitochondrial dysfunction in the placenta and maternal endothelium [89,90]. This mitochondrial dysfunction is associated with impaired electron transport chain activity, leading to excessive ROS generation, particularly at complexes I and III [78,91]. The Nrf2/Keap1 pathway plays a crucial role in regulating the cellular response to oxidative stress. Nrf2, a transcription factor, promotes the expression of antioxidant proteins that restore redox balance [92,93]. Studies have shown that in preeclamptic placentas, Nrf2 expression is significantly upregulated in response to oxidative stress, while the activity of oxidative stress-related enzymes such as superoxide dismutase (SOD), catalase (CAT), and glutathione peroxidase (GSH-Px) is significantly reduced [94,95]. 

#### 2.3.4. Endothelial Cell Activation

There is an increased expression of adhesion molecules such as intercellular adhesion molecule-1 (ICAM-1) and vascular cell adhesion molecule-1 (VCAM-1) by the endothelial cells, promoting leukocyte adhesion and transmigration [64]. This leukocyte infiltration into the vascular wall contributes to a pro-inflammatory and pro-thrombotic state, which is characteristic of PE [58]. The increased shedding of syncytiotrophoblast microparticles (STBM) into the maternal circulation, following trophoblast apoptosis, also plays a role in systemic inflammation and endothelial dysfunction [58,96].

#### 2.3.5. The Role of Adipokines

Adipokines like chemerin and leptin are significantly elevated in PE and contribute to endothelial dysfunction and metabolic dysregulation [65,66]. Chemerin, highly expressed in placental tissue, influences lipid metabolism, glucose homeostasis, and inflammation [65]. It promotes ROS production, endothelial cell autophagy, and vascular smooth muscle cell (VSMC) dysfunction. Elevated chemerin levels are associated with increased arterial stiffness and impaired endothelial function [65,67,97]. Leptin, another key adipokine, is elevated in PE and linked to placental hypoxia and systemic inflammation. Leptin affects angiogenesis, contributing to the imbalance between pro- and anti-angiogenic factors [66].

#### 2.3.6. Systemic Inflammation

Systemic inflammation in PE is characterized by elevated levels of pro-inflammatory cytokines such as tumor necrosis factor-alpha (TNF-α) and interleukin-6 (IL-6). These cytokines are produced in response to placental ischemia and hypoxia, leading to an inflammatory response that further damages the endothelium [68]. The activation of inflammatory cells, including NK cells and CD4+ T cells, contributes to the release of ROS and pro-inflammatory mediators, perpetuating the cycle of endothelial damage and oxidative stress [89]. Both NF-κB and JNK, are upregulated in preeclampsia, leading to increased production of pro-inflammatory cytokines. Systemic inflammation in PE involves chronic immune activation and elevated levels of inflammatory cytokines such as TNF-α, IL-6, and IL-17 [31]. These cytokines promote endothelial dysfunction by increasing the expression of adhesion molecules and reducing NO bioavailability, leading to vasoconstriction and hypertension [31]. The pro-inflammatory state in PE is further characterized by an imbalance in T-helper cell subsets, with increased Th1 and Th17 cells and decreased regulatory T cells (Tregs) [69,98,99,100]. The reduction in Tregs and their associated cytokine IL-10 contributes to the inflammatory milieu and impaired immune regulation in PE [31].

#### 2.3.7. The Role of Ferroptosis

Ferroptosis, a form of iron-dependent cell death, has also been implicated in the pathogenesis of PE. Ferroptosis is driven by the accumulation of lipid peroxides and is regulated by the activity of glutathione peroxidase 4 (GPX4) and the availability of iron [70,101]. In PE, dysregulation of iron metabolism and increased lipid peroxidation contribute to ferroptosis in placental trophoblasts, leading to placental dysfunction and exacerbating oxidative stress [102,103].

Recent studies have highlighted the role of lipid metabolism in ferroptosis, showing that polyunsaturated fatty acids (PUFAs) are particularly susceptible to peroxidation, leading to ferroptotic cell death [71,72,104]. In PE, the abundance of iron in trophoblasts and the dysregulation of lipid metabolism promote ferroptosis, contributing to placental and endothelial dysfunction.

#### 2.3.8. Genetic and Epigenetic Factors

There are genetic and epigenetic factors involved in the physiopathology of PE (Table 3). For instance, polymorphisms within the methylenetetrahydrofolate reductase (MTHFR) gene, specifically the hypomethylation of its promoter region, are associated with increased plasma homocysteine levels and decreased MTHFR enzyme activity [75]. Epigenetic modifications, such as DNA methylation and microRNA (miRNA) expression, also play a vital role. The hypomethylation of the MTHFR gene promoter leads to its overexpression, which is linked to elevated homocysteine levels, further implicating endothelial dysfunction in the disease’s pathogenesis [73]. Additionally, the upregulation of miR-155 in the placenta is another crucial epigenetic change observed in preeclampsia. MiR-155, which can be significantly induced by inflammatory stimuli like tumor necrosis factor-alpha (TNF-α) and lipopolysaccharides, regulates genes involved in angiogenesis and endothelial function. This miRNA targets and downregulates cysteine-rich angiogenic inducer 61 (CYR61), a key factor in angiogenesis, leading to reduced vascularization and placental insufficiency [74].

Genetic variations in the FLT1 gene also contribute to PE. Variants such as rs4769613 and rs12050029 have been associated with preeclampsia, though not all studies find significant differences in genotype frequencies between preeclamptic and normotensive pregnancies. The rs149427560 variant in the upstream region of FLT1 has been shown to affect the expression of the FLT1 gene, leading to increased production of anti-angiogenic factors like sFlt-1, which are central to the abnormal vascular remodeling seen in PE [76].

Furthermore, the polymorphism rs767649 in the miR-155 promoter region enhances its expression, exacerbating its effects on gene regulation and inflammation. The presence of the A allele in this polymorphism significantly increases the risk of PE, demonstrating how genetic variants influence epigenetic regulation and disease susceptibility [105].

The complement system, particularly the C3 gene, illustrates the role of epigenetics in PE. Specific single nucleotide polymorphisms (SNPs) in the C3 gene, such as rs2287845, affect gene expression through mechanisms involving epigenetic regulation. These polymorphisms can influence splicing and the overall function of the C3 protein, contributing to the inflammatory and immune responses characteristic of severe PE [106].

Polymorphisms in the TNF-alpha gene, especially at positions −308 and −238, are also critical. The G/G genotype at the −308 position is more frequent in preeclamptic women and is associated with higher TNF-alpha expression, promoting inflammation and endothelial dysfunction. These genetic variations underscore the role of TNF-alpha in the disease’s pathogenesis and highlight how genetic and epigenetic interactions influence the severity and occurrence of PE [107].

Alternative splicing of the sFlt-1 gene is another key epigenetic mechanism in PE. sFlt-1, a splice variant of the vascular endothelial growth factor receptor 1 (VEGFR-1), acts as an anti-angiogenic factor by binding to VEGF and PlGF, preventing them from interacting with their receptors on the endothelial surface. Increased production of sFlt-1 in PE, driven by hypoxia and other stress signals in the placenta, leads to reduced angiogenesis and endothelial dysfunction. This alternative splicing results in a soluble form of sFlt-1 that is released into the maternal circulation, contributing to the clinical manifestations of PE, such as hypertension and proteinuria [32].

Long noncoding RNAs (lncRNAs), such as maternally expressed gene 3 (MEG3), play a vital role in PE. MEG3 is underexpressed in preeclamptic placentas and its downregulation decreases migration and invasion while promoting apoptosis of trophoblast cells [108,109]. MEG3 improves trophoblast function by regulating the Wnt/β-Catenin/NLRP3 pathway, which is crucial for cell proliferation, migration, and invasion, and by reducing inflammation [110]. Upregulation of MEG3 has been shown to enhance trophoblast cell function and alleviate PE progression.

Additionally, the NO signaling pathway is essential for vascular function and is influenced by genetic and epigenetic factors. The endothelial NO synthase (NOS3) gene and the guanylate cyclase 1, soluble, alpha 3 (GUCY1A3) gene are central to NO production and signaling. Polymorphisms in these genes, such as NOS3 rs3918226 and GUCY1A3 rs7692387, affect NO bioavailability. Reduced NO production due to these genetic variations leads to endothelial dysfunction, a critical aspect of PE [111].

**Table 3:** Genetic and epigenetic factors in preeclampsia.

Genetic/Epigenetic Factor	Description	Effect	Key Gene/Factor
MTHFR polymorphisms	Hypomethylation of MTHFR promoter increases homocysteine levels and reduces enzyme activity [73,75].	Contributes to endothelial dysfunction.	MTHFR
miR-155 upregulation	Induced by inflammatory stimuli (TNF-α, LPS); regulates genes involved in angiogenesis [74].	Leads to reduced vascularization and placental insufficiency.	miR-155, CYR61
FLT1 gene variants	Polymorphisms (e.g., rs4769613, rs12050029, rs149427560) affect FLT1 expression [76].	Increases sFlt-1 production, disrupting vascular remodeling.	FLT1, sFlt-1
miR-155 promoter polymorphism	rs767649 polymorphism enhances miR-155 expression [105].	Heightens inflammation and gene regulation disruption.	miR-155
Complement system SNPs	SNPs in the C3 gene (e.g., rs2287845) affect gene expression through epigenetic regulation [106].	Contributes to inflammatory and immune responses.	C3
TNF-α gene polymorphisms	Polymorphisms at -308 and -238 positions increase TNF-α expression [107].	Promotes inflammation and endothelial dysfunction.	TNF-α
sFlt-1 alternative splicing	Hypoxia induces alternative splicing of the sFlt-1 gene [32].	Increases soluble sFlt-1, reducing angiogenesis and causing endothelial dysfunction.	sFlt-1
MEG3 downregulation	lncRNA MEG3 is underexpressed in preeclamptic placentas [108].	Decreases trophoblast migration and invasion, increases apoptosis.	MEG3
NOS3 and GUCY1A3 polymorphisms	Polymorphisms affecting NO production and signaling [111].	Leads to reduced NO bioavailability and endothelial dysfunction.	NOS3, GUCY1A3

MTHFR: methylenetetrahydrofolate reductase; miR-155: microRNA 155; FLT1: fms-like tyrosine kinase 1; sFlt-1: soluble fms-like tyrosine kinase-1; TNF-α: tumor necrosis factor-alpha; C3: complement component 3; MEG3: maternally expressed gene 3; NOS3: nitric oxide synthase 3; GUCY1A3: guanylate cyclase 1, soluble, alpha 3; NO: nitric oxide; LPS: lipopolysaccharides; CYR61: cysteine-rich angiogenic inducer 61.

#### 2.3.9. Other Associated Mechanisms

PE is influenced by various other mechanisms, including mitochondrial dysfunction, platelet activation, RAAS dysregulation, oxidative stress, micronutrient deficiencies, and key protective pathways such as HO-1/CO and CSE/H2S. These factors collectively exacerbate oxidative stress, inflammation, and endothelial dysfunction, contributing to the complex pathophysiology of the disease. Table 4 summarizes these mechanisms and their effects.

#### 2.3.10. The Role of Mitochondrial Dysfunction

Studies have demonstrated that women with preeclampsia exhibit abnormalities in mitochondrial gene expression and increased mitochondrial lipid peroxidation, leading to heightened oxidative stress. Coenzyme Q10 (CoQ10), a vital component of the mitochondrial respiratory chain, plays a critical role in energy production and ROS formation. In PE, CoQ10 levels are significantly reduced, likely due to increased ROS production, exacerbating oxidative stress and endothelial dysfunction [33,34,78].

#### 2.3.11. The Role of Platelet Activation

Platelets are essential for hemostasis and the maternal thrombo-inflammatory response characteristic of PE [77]. In patients with PE, heightened platelet activation is evidenced by increased mean platelet volume, generation of platelet microparticles, and expression of activation markers such as P-selectin and CD63 [123]. This hyperactivation contributes to the prothrombotic state observed in PE. Interestingly, platelets in PE are hyperactivated yet exhibit impaired aggregation in response to agonists like ADP and collagen, indicating a complex dysregulation of platelet function [77]. Extracellular vesicles (EVs) derived from activated platelets and other cells further contribute to PE by triggering inflammasome activation and promoting a pro-inflammatory state [77,123].

#### 2.3.12. The Role of the Renin-Angiotensin-Aldosterone System

Overexpression of angiotensin II and its vasoconstrictive effects contribute to hypertension and endothelial dysfunction in PE [79]. Angiotensin II induces low-grade inflammation in endothelial and vascular cells, further perpetuating the pro-inflammatory state. Additionally, the dysregulation of RAAS affects blood pressure regulation, contributing to the hypertensive manifestations of PE.

#### 2.3.13. The Role of Oxidative Stress

Oxidative stress biomarkers and angiogenic growth mediators (AGMs) are crucial in understanding preeclampsia’s pathophysiology. The initial stage involves syncytiotrophoblast damage and local placental hypoxia, leading to increased oxidative stress [82]. This stage is characterized by changes in circulating sFlt-1 and PlGF levels [112]. The subsequent stage involves the synthesis and release of vasoactive factors into maternal circulation, the unusual expression of pro-inflammatory cytokines, dysregulation in AGMs, and endothelial cell dysfunction [113]. Elevated levels of 8-hydroxydeoxyguanosine (8-OHdG) in preeclamptic women are markers of oxidative stress and DNA damage. This oxidative stress is associated with increased serum levels of sFlt-1, 8-epi-prostaglandin F2α (8-epi-PGF2α), and decreased levels of PlGF and total antioxidant capacity (TAC) [112,124]. This imbalance in AGMs and oxidative stress biomarkers leads to detrimental pregnancy outcomes such as stillbirth, intrauterine growth restriction (IUGR), and postpartum hemorrhage.

#### 2.3.14. The Role of Micronutrient Deficiencies and Inositol Messengers

Micronutrient deficiencies, such as calcium and magnesium, also play a significant role in modulating oxidative stress and angiogenic balance in PE. Deficiencies in these micronutrients correlate with imbalanced AGMs and oxidative stress biomarkers, which are more prominent in early-onset preeclampsia (EO-PE), diagnosed before 34 weeks of gestation, compared to late-onset preeclampsia (LO-PE), diagnosed after 34 weeks of gestation [82].

Inositol messengers, particularly inositol phosphoglycans (IPG-P and IPG-A), are key regulators of the pyruvate dehydrogenase complex (PDC). PDC is the interface between glycolysis and the citric acid cycle, crucial for generating ATP, acetyl-CoA, and NADH by mitochondria. IPGs modulate PDC activity through effects on PDK and PDP [83]. These phosphoglycans, containing trace metals like zinc and manganese, play a significant role in regulating metabolic pathways, potentially contributing to the metabolic disturbances observed in PE.

#### 2.3.15. The Role of HO-1/CO and CSE/H2S Pathways

The heme oxygenase-1 (HO-1)/carbon monoxide (CO) and cystathionine gamma-lyase (CSE)/hydrogen sulfide (H2S) pathways also play protective roles in PE. HO-1 and CO inhibit the release of anti-angiogenic factors such as sFlt-1 and sEng. Women with PE have lower HO-1 expression and exhale less CO, suggesting a loss of HO activity contributes to the disease’s pathogenesis. Statins, which stimulate HO-1 expression and inhibit sFlt-1 release, show potential in ameliorating PE [80].

Hydrogen sulfide (H2S), produced mainly by cystathionine gamma-lyase (CSE), is a pro-angiogenic vasodilator. Decreased CSE activity in preeclampsia leads to reduced H2S levels, contributing to abnormal placentation and maternal hypertension [81,125]. Inhibition of CSE activity results in decreased PlGF production and impaired trophoblast invasion, further exacerbating preeclampsia’s pathophysiology. Restoring H2S levels through slow-releasing H2S-generating compounds has shown promise in reducing sFlt-1 and sEng levels and improving fetal growth in animal models [80].

#### 2.3.16. The Role of Environmental Factors and Endocrine Disruptors

In addition to the genetic, molecular, and immunological factors, environmental agents, particularly endocrine disruptors, play a significant role in the pathogenesis of PE. Endocrine disruptors such as bisphenol A (BPA) and phthalates, found in plastics, personal care products, and other consumer goods, can interfere with hormone function and have been linked to adverse cardiovascular and reproductive outcomes [116].

Recent studies have highlighted the impact of BPA and phthalates on prenatal cardiovascular health and their potential role in PE. BPA, for instance, has been shown to alter placental function by disrupting the estrogen and androgen signaling pathways [116,118]. This disruption affects the expression of genes such as estrogen receptor 1 (ESR1), which is crucial for placental vascularization and trophoblast invasion. Additionally, BPA induces oxidative stress by increasing the production of ROS, leading to endothelial dysfunction and inflammation—a key feature of PE [114,116]. Genes involved in oxidative stress response, such as nuclear factor erythroid 2-related factor 2 (NFE2L2), are often upregulated in response to BPA exposure, indicating a compensatory mechanism that may become overwhelmed in the context of PE [115,126].

Phthalates, on the other hand, are known to affect lipid metabolism and inflammatory responses. They interfere with the peroxisome proliferator-activated receptor gamma (PPARγ) signaling pathway, which is vital for lipid homeostasis and anti-inflammatory responses [115]. Disruption of PPARγ signaling can lead to altered expression of genes involved in lipid metabolism, such as fatty acid binding protein 4 (FABP4) and CD36, contributing to the development of a pro-inflammatory state and endothelial dysfunction. Phthalates have also been shown to increase the expression of inflammatory cytokines like TNF-α and IL-6, which are implicated in the pathogenesis of PE [115,127].

Furthermore, both BPA and phthalates can cause epigenetic modifications that affect gene expression related to placental and vascular function. For example, BPA exposure has been linked to hypomethylation of the promoter regions of genes involved in angiogenesis, such as VEGF and its receptors (FLT1 and KDR), leading to impaired placental development and increased susceptibility to PE [117,119].

#### 2.3.17. The Role of Estrogens and Androgens

Estrogens and androgens play crucial roles in the pathophysiology of PE by significantly affecting placental development and maternal vascular function [122]. Estrogens, primarily through their interaction with ESR1 and ESR2, are essential for maintaining pregnancy by promoting vasodilation and increasing NO synthesis via upregulation of endothelial nitric oxide synthase (eNOS) [128]. This vasodilatory effect is crucial for reducing vascular resistance and ensuring adequate blood flow to the placenta. In PE, disrupted estrogen signaling impairs NO production, leading to endothelial dysfunction and heightened vascular resistance [128].

Estrogens regulate the balance of angiogenic factors, such as VEGF and PlGF, which are vital for angiogenesis and placental development. Estrogens enhance the expression of VEGF and PlGF by binding to estrogen receptors (ERs) on endothelial cells and trophoblasts, which activates the transcription of genes encoding these angiogenic factors. VEGF promotes the growth and formation of new blood vessels, while PlGF supports placental vascular development and trophoblast invasion. By upregulating VEGF and PlGF, estrogens facilitate proper placental perfusion and oxygenation, which are crucial for fetal development [129]. In PE, impaired estrogen signaling can disrupt this balance, leading to reduced expression of VEGF and PlGF, contributing to placental hypoxia and oxidative stress, which are central to PE pathogenesis.

Androgens, such as testosterone and dihydrotestosterone (DHT), influence PE development through their effects on vascular function and angiogenic factor balance. Elevated androgen levels, often observed in preeclamptic women, exert their effects through androgen receptors (AR) in the placenta and maternal vasculature. Increased androgen levels can induce vasoconstriction and endothelial dysfunction by decreasing NO bioavailability and enhancing oxidative stress via upregulation of NADPH oxidase, which increases ROS production [120].

Furthermore, androgens alter the balance of angiogenic factors by promoting the expression of anti-angiogenic molecules such as sFlt-1 and sEng. The mechanism behind this involves the androgen-mediated regulation of gene expression related to angiogenesis. Androgens can upregulate the expression of the sFlt-1 and sEng genes in the placenta, leading to higher levels of these proteins in the maternal circulation. Elevated levels of sFlt-1 act as decoy receptors for VEGF and PlGF, binding these pro-angiogenic factors and preventing them from interacting with their receptors on the endothelial surface. This disruption in signaling leads to impaired angiogenesis and endothelial repair, contributing to placental hypoxia and systemic endothelial dysfunction. Similarly, elevated sEng levels interfere with the TGF-β signaling pathway, further exacerbating endothelial dysfunction and inflammation [120,121].

Additionally, both hormones influence immune responses and inflammatory pathways. Estrogens modulate immune cell activity, including NK cells and Tregs, which are vital for proper trophoblast invasion and placental development. Disrupted estrogen-mediated immune regulation can lead to inadequate spiral artery remodeling and placental insufficiency, key features of PE. Androgens affect lipid metabolism and inflammatory responses by altering the PPARγ signaling pathway. Disruption of PPARγ signaling leads to altered expression of genes involved in lipid metabolism, such as FABP4 and CD36, contributing to a pro-inflammatory state [130]. Elevated androgen levels are associated with increased expression of inflammatory cytokines like TNF-α and IL-6, further exacerbating endothelial dysfunction and inflammation in PE [131].

## 3. Discussion

### 3.1. Main Findings

This narrative review explores the complex and multifactorial pathogenesis of PE, focusing on two primary stages: abnormal placentation and maternal systemic response. The first stage involves insufficient trophoblastic invasion and inadequate remodeling of the spiral arteries, resulting in poor placental perfusion and hypoxia. This placental dysfunction leads to the release of antiangiogenic factors such as sFlt-1 and sEng, which contribute to endothelial dysfunction and systemic inflammation. Genetic predispositions and epigenetic modifications further influence the susceptibility and severity of PE. Additionally, environmental agents, particularly endocrine disruptors like BPA and phthalates, are emerging as significant factors in PE pathogenesis due to their impact on hormonal regulation and cardiovascular health.

### 3.2. Clinical Interpretation

Understanding the multifactorial nature of PE, including the influence of genetic, environmental, and hormonal factors, has significant clinical implications. Early diagnosis and risk stratification can be enhanced by measuring levels of antiangiogenic factors like sFlt-1 and sEng. These biomarkers can help identify patients at risk for PE, allowing for timely intervention. Potential therapeutic strategies, such as low-dose aspirin and statins, which improve endothelial function, offer promise in reducing the incidence and severity of preeclampsia. The role of environmental contaminants underscores the importance of public health measures to reduce exposure to endocrine disruptors, potentially mitigating some risk factors for PE. 

Addressing the complexity of PE requires a multidisciplinary approach involving gynecologists, internists specialized in obstetric medicine, nephrologists, cardiologists, intensivists, and other specialists [132]. This collaboration can uncover new phenotypes and research directions, enhancing our understanding of preeclampsia and improving patient outcomes. Integrating diverse expertise aligns with recommendations for optimizing healthcare in pregnancy-related conditions, ultimately aiming to reduce maternal and fetal morbidity and mortality.

### 3.3. Potential Therapeutic Targets

Understanding the pathophysiology of PE has opened avenues for potential therapeutic interventions. One of the promising areas of investigation is targeting oxidative stress, which is known to play a crucial role in the development of PE. Antioxidants such as vitamins C and E have been studied extensively for their potential to reduce oxidative stress and improve maternal and fetal outcomes [133]. Some clinical trials suggest that these vitamins can reduce oxidative stress and improve maternal and fetal outcomes [134,135]. However, other studies have not demonstrated significant benefits, highlighting variability in results that may be due to differences in study designs, antioxidant doses, and the gestational age at which treatment is initiated [133,136].

Modulating the inflammatory response is another therapeutic strategy. Statins, known for their cholesterol-lowering effects, also possess anti-inflammatory and antioxidant properties. These drugs can improve endothelial function and have shown promise in reducing the severity of PE [137]. Similarly, low-dose aspirin has been widely recommended for women at high risk of developing PE. Studies have demonstrated that early administration of low-dose aspirin can significantly reduce the incidence of PE, likely by modulating inflammatory pathways and improving placental blood flow [138].

A critical aspect of PE pathogenesis is the imbalance between pro-angiogenic and anti-angiogenic factors. Therapeutic strategies aiming to restore this balance are currently under investigation. Recombinant VEGF and PlGF are being studied for their potential to enhance placental perfusion and reduce endothelial dysfunction [139,140]. These angiogenic therapies offer a promising approach to addressing the root causes of PE, though further research is necessary to determine their safety and efficacy in pregnant women.

In addition to these targeted therapies, broader approaches to improving maternal health, such as managing preexisting conditions like hypertension and diabetes, optimizing maternal nutrition, and ensuring early and regular prenatal care, are essential components of a comprehensive strategy to prevent and treat PE. The complexity of preeclampsia’s pathophysiology underscores the need for multifaceted therapeutic approaches. By continuing to explore and refine these strategies, we can improve maternal and fetal outcomes and reduce the burden of this challenging condition.

### 3.4. Research Interpretation and Future Directions

Future research should focus on clarifying the molecular mechanisms linking abnormal placentation to maternal systemic responses. Longitudinal studies are needed to monitor the progression of PE and identify critical windows for intervention. Additionally, research should continue to explore the genetic, epigenetic, and environmental bases of PE to uncover new therapeutic targets. Investigating the efficacy and safety of angiogenic therapies, which aim to restore placental perfusion and endothelial function, is a promising avenue. The role of ion channels in trophoblast invasion and vascular remodeling is also crucial. Ion channels, including calcium, potassium, and sodium channels, are essential for proper placental function and fetal development. Abnormal regulation of these channels can contribute to the pathogenesis of PE. Future studies should explore how modulation of ion channels can be targeted for therapeutic interventions. A multidisciplinary approach combining clinical, genetic, environmental, and molecular research will be essential in advancing our understanding and management of PE. By addressing these various aspects, we can develop more effective strategies to prevent and treat preeclampsia, ultimately improving outcomes for both mothers and their babies.

## 4. Materials and Methods

### 4.1. Eligibility Criteria

We conducted a systematic search using specific MeSH terms related to PE, including “preeclampsia”, “pathophysiology”, “pathogenesis”, “abnormal placentation”, “endothelial dysfunction”, “systemic inflammation”, and “oxidative stress”. Detailed descriptions of the search strategy and the specific query syntaxes used can be found in Table S1.

The inclusion criteria for this review were original studies on basic and clinical science, systematic reviews, and narrative reviews that focused on the pathophysiology of PE. 

This scoping review adhered to the PRISMA extension for scoping reviews [141] (Figure S1). 

### 4.2. Study Selection

Abstracts identified as relevant were assessed by two independent evaluators (J.T.-T. and L.R.-Z.). This initial screening was conducted without knowledge of the articles’ authorship, author affiliations, or study results to minimize bias. Articles that appeared relevant based on their titles and abstracts were selected for a more detailed review. In the second stage, the preselected articles were read in their entirety to ensure they met all the inclusion criteria. 

### 4.3. Data Extraction and Analysis

Data extraction from the selected articles was performed using a standardized form designed to capture essential study characteristics. This form included details such as the author, year of publication, country, and study design. Additionally, it captured specific details related to the pathophysiology of PE, key findings, and conclusions drawn from each study.

The results obtained from the selected articles were then analyzed and organized for presentation in relevant sections of this review. The most significant findings were highlighted, and connections were established between different approaches to understanding the pathophysiology of PE and potential therapeutic targets. This systematic approach ensured that the review provided a thorough and nuanced understanding of the current state of research on PE pathophysiology (Table S2).

## 5. Conclusions

Preeclampsia is a complex disorder with multifactorial origins involving abnormal placentation, endothelial dysfunction, systemic inflammation, and oxidative stress. Despite advances in understanding its pathophysiology, effective prevention and treatment strategies remain limited. Continued research is essential to develop targeted therapies that can improve outcomes for both mothers and their babies.

## Figures and Tables

**Table 1:** Mechanisms associated with the pathogenesis of preeclampsia.

Associated Mechanisms	Description
Abnormal placentation	Early pregnancy issues involving deficient invasion of extravillous trophoblasts and inadequate remodeling of spiral arteries, leading to reduced placental perfusion and hypoxia. Factors include genetic predisposition, reduced HLA-G expression, and dysfunctional interactions between uterine NK cells and trophoblasts [27,28,29].
Maternal systemic response	Endothelial dysfunction caused by the hypoxic placenta-releasing factors like sFlt-1 and sEng, resulting in increased vascular permeability and heightened vascular reactivity. Clinical manifestations include hypertension and proteinuria due to an imbalance between vasodilators (nitric oxide) and vasoconstrictors (endothelin-1) [30].
Systemic inflammation and oxidative stress	Oxidative stress from excess reactive oxygen species (ROS) production due to placental hypoxia, damaging endothelial cells. Systemic inflammation involves elevated pro-inflammatory cytokines (TNF-α, IL-6), activating inflammatory signaling pathways, and perpetuating endothelial damage and oxidative stress [31].
Genetic and epigenetic factors	Genetic variants include polymorphisms in genes regulating endothelial function, inflammatory response, and angiogenesis (e.g., VEGF, ENG) that increase preeclampsia risk. Epigenetic modifications involve DNA methylation and histone modifications affecting key genes involved in blood pressure regulation and placental function [32].
Other associated mechanisms	Mitochondrial dysfunction leads to excessive ROS production and oxidative damage, exacerbating endothelial dysfunction. Inflammatory pathways, such as NF-κB and JNK, increase pro-inflammatory cytokine production. RAAS alterations include overexpression of angiotensin II, contributing to hypertension [33,34].

HLA-G: human leukocyte antigen-G; NK: natural killer; sFlt-1: soluble fms-like tyrosine kinase-1; sEng: soluble endoglin; TNF-α: tumor necrosis factor-alpha; IL-6: interleukin-6; VEGF: vascular endothelial growth factor; ENG: endoglin; ROS: reactive oxygen species; NF-κB: nuclear factor kappa-light-chain-enhancer of activated B cells; JNK: c-Jun N-terminal kinase; RAAS: renin-angiotensin-aldosterone system.

**Table 2:** Mechanisms and effects in the maternal systemic response of preeclampsia.

Mechanism	Description	Effect	Key Substance
Pro-angiogenic and anti-angiogenic factors	Inadequate trophoblast invasion and poor spiral artery remodeling trigger overproduction of sFlt-1 and sEng [59].	Disruption of angiogenesis and endothelial repair, resulting in reduced NO production and clinical manifestations of PE [60]. sFlt-1 binds and neutralizes VEGF and PlGF, disrupting angiogenesis and endothelial repair, leading to endothelial dysfunction [30]. sEng inhibits TGF-β signaling, exacerbating endothelial damage [30,59].	sFlt-1, sEng, VEGF, PlGF, TGF-β, CD93
Endothelial dysfunction	Characterized by increased vascular permeability and reactivity, disrupted balance between vasodilators and vasoconstrictors [60]	Leads to vasoconstriction, increased blood pressure, and a pro-inflammatory state [59,60].	NO, endothelin-1, ROS, TNF-α, IL-6
Oxidative stress	Hypoxic placenta generates excess ROS through NADPH oxidase and xanthine oxidase activities [61].	ROS damage cellular components, leading to endothelial cell apoptosis and further dysfunction [62,63].	ROS, NADPH oxidase, xanthine oxidase
Endothelial cell activation	Increased expression of adhesion molecules by endothelial cells [64].	Promotes leukocyte adhesion and transmigration, contributing to a pro-inflammatory and pro-thrombotic state [59,60].	ICAM-1, VCAM-1
The role of adipokines	Elevated levels of chemerin and leptin in PE [65].	Contributes to endothelial dysfunction, metabolic dysregulation, and increased ROS production [66,67].	Chemerin, leptin
Systemic inflammation	Characterized by elevated levels of pro-inflammatory cytokines [68].	Promotes endothelial dysfunction, increased ROS, and chronic immune activation [69].	TNF-α, IL-6, IL-17, Th1, Th17, Tregs, IL-10
The role of ferroptosis	Iron-dependent cell death driven by lipid peroxidation and dysregulated iron metabolism [70].	Contributes to placental dysfunction and exacerbates oxidative stress [71,72].	GPX4, iron, PUFAs
Epigenetic factors	Hypomethylation and upregulation of specific genes like MTHFR and miR-155 [73].	Altered gene expression leading to endothelial dysfunction and inflammation [74].	MTHFR, miR-155
Genetic factors	Polymorphisms in genes affecting angiogenesis and NO bioavailability [75]	Contributes to endothelial dysfunction and hypertension [75,76].	sFlt-1, NOS3, GUCY1A3
Other associated factors	Mitochondrial dysfunction, platelet activation, renin-angiotensin-aldosterone system, and adipokines [77,78,79].	Contribute to oxidative stress, endothelial dysfunction, and inflammation [77,78,79].	CoQ10, platelets, angiotensin II
Role of HO-1/CO and CSE/H2S pathways	Protective roles in preeclampsia by inhibiting the release of anti-angiogenic factors [80].	Loss of HO activity contributes to pathogenesis, while H2S acts as a pro-angiogenic vasodilator [80,81].	HO-1, CO, CSE, H2S
Micronutrient deficiencies and inositol messengers	Calcium and magnesium deficiencies modulate oxidative stress and angiogenic balance, while inositol messengers regulate the pyruvate dehydrogenase complex [82].	Micronutrient deficiencies correlate with imbalanced AGMs and oxidative stress biomarkers, contributing to metabolic disturbances [82,83].	Calcium, magnesium, IPG-P, IPG-A

sFlt-1: soluble fms-like tyrosine kinase-1; sEng: soluble endoglin; VEGF: vascular endothelial growth factor; PlGF: placental growth factor; TGF-β: transforming growth factor-beta; NO: nitric oxide; ROS: reactive oxygen species; TNF-α: tumor necrosis factor-alpha; IL-6: interleukin-6; ICAM-1: intercellular adhesion molecule-1; VCAM-1: vascular cell adhesion molecule-1; IL-17: interleukin-17; Th1: T-helper 1 cells; Th17: T-helper 17 cells; Tregs: regulatory T cells; GPX4: glutathione peroxidase 4; PUFAs: polyunsaturated fatty acids; MTHFR: methylenetetrahydrofolate reductase; NOS3: nitric oxide synthase 3; GUCY1A3: guanylate cyclase 1 soluble alpha 3; CO: carbon monoxide; CSE: cystathionine gamma-lyase; H2S: hydrogen sulfide; IPG-P: inositol phosphoglycan-P; IPG-A: inositol phosphoglycan-A.

**Table 4:** Other associated mechanisms in preeclampsia.

Mechanism	Description	Effect	Key Factors
Mitochondrial dysfunction	Abnormalities in mitochondrial gene expression and lipid peroxidation [78].	Increased oxidative stress and endothelial dysfunction.	Mitochondrial genes, CoQ10
Platelet activation	Increased activation markers (e.g., P-selectin, CD63) and mean platelet volume [77].	Contributes to prothrombotic state and inflammation.	P-selectin, CD63, platelets
RAAS dysregulation	Overexpression of angiotensin II and its vasoconstrictive effects [79].	Leads to hypertension and endothelial dysfunction.	Angiotensin II, RAAS
Oxidative stress	Syncytiotrophoblast damage and local hypoxia increase ROS production [112,113].	Leads to DNA damage, impaired angiogenesis, and adverse outcomes.	ROS, sFlt-1, PlGF, 8-OHdG
Micronutrient deficiencies	Deficiencies in calcium and magnesium [82].	Modulates oxidative stress and angiogenic balance.	Calcium, magnesium
Inositol messengers	Regulation of the pyruvate dehydrogenase complex by IPG-P and IPG-A [83].	Contributes to metabolic disturbances in preeclampsia.	IPG-P, IPG-A
HO-1/CO pathway	HO-1 and CO inhibit release of anti-angiogenic factors [80].	Protective role by reducing sFlt-1 and sEng levels.	HO-1, CO
CSE/H2S pathway	H2S produced by CSE acts as a pro-angiogenic vasodilator [81].	Reduced H2S leads to abnormal placentation and hypertension.	CSE, H2S
Environmental factors and endocrine disruptors	Exposure to compounds like BPA and phthalates disrupts hormone signaling and induces oxidative stress [114,115,116,117,118,119].	Leads to impaired placental function and increased risk of preeclampsia.	BPA, phthalates, ESR1, PPARγ, TNF-α, IL-6, VEGF
Estrogen and androgen dysregulation	Disrupted estrogen and androgen signaling affects vascular function and angiogenic balance [120,121,122].	Leads to endothelial dysfunction, impaired placental development, and inflammation.	ESR1, ESR2, eNOS, VEGF, PlGF, AR, NADPH oxidase, sFlt-1, sEng, PPARγ, TNF-α, IL-6

ROS: reactive oxygen species; RAAS: renin-angiotensin-aldosterone system; sFlt-1: soluble fms-like tyrosine kinase-1; PlGF: placental growth factor; 8-OHdG: 8-hydroxydeoxyguanosine; CoQ10: coenzyme Q10; IPG-P: inositol phosphoglycan-P; IPG-A: inositol phosphoglycan-A; HO-1: heme oxygenase-1; CO: carbon monoxide; CSE: cystathionine gamma-lyase; H2S: hydrogen sulfide; BPA: bisphenol A; ESR1: estrogen receptor 1; PPARγ: peroxisome proliferator-activated receptor gamma; TNF-α: tumor necrosis factor-alpha; IL-6: interleukin-6; VEGF: vascular endothelial growth factor; ESR1: estrogen receptor 1; ESR2: estrogen receptor 2; eNOS: endothelial nitric oxide synthase; AR: androgen receptor; NADPH: nicotinamide adenine dinucleotide phosphate.

## Data Availability

The data employed for conducting this narrative review are available upon request to the following e-mails: torresmmf@gmail.com; salvadorespino@gmail.com.

## Supplementary Materials

The supporting information can be downloaded at: https://www.mdpi.com/article/10.3390/ijms25147569/s1.
