# Supplementary material for: A Narrative Review on the Pathophysiology of Preeclampsia
Source: Int J Mol Sci. 2024 Jul 10;25(14):7569. doi: 10.3390/ijms25147569 (PMC11277207; doi:10.3390/ijms25147569)
Supplement: Supplementary file 1 [file ijms-25-07569-s001.zip › ijms-3083862-supplementary.pdf]

Table S1. Search strategies	
**PubMed**	Preeclampsia AND (pathophysiology OR pathogenesis OR "abnormal placentation" OR "endothelial dysfunction" OR "systemic inflammation" OR "oxidative stress") AND ("2012/05/20"[Date - Publication] : "2024/05/20"[Date - Publication]) NOT (animals[MeSH Terms] OR mice[Title] OR rats[Title])<br><br>Filters: English language, human studies.
**MEDLINE**	Preeclampsia AND (pathophysiology OR pathogenesis OR "abnormal placentation" OR "endothelial dysfunction" OR "systemic inflammation" OR "oxidative stress") AND (2012-2024) NOT (animals OR mice OR rats)<br><br>Filters: English language, human studies, review articles.
**Google Scholar**	"Preeclampsia" AND (pathophysiology OR pathogenesis OR "abnormal placentation" OR "endothelial dysfunction" OR "systemic inflammation" OR "oxidative stress") AND 2012..2024 -animals -mice -rats<br><br>Filters: Articles published in the last 10 years, relevance.
**Scopus**	TITLE-ABS-KEY(preeclampsia) AND (TITLE-ABS-KEY(pathophysiology) OR TITLE-ABS-KEY(pathogenesis) OR TITLE-ABS-KEY("abnormal placentation") OR TITLE-ABS-KEY("endothelial dysfunction") OR TITLE-ABS-KEY("systemic inflammation") OR TITLE-ABS-KEY("oxidative stress")) AND PUBYEAR > 2012 AND PUBYEAR < 2025 AND NOT (TITLE-ABS-KEY(mice) OR TITLE-ABS-KEY(rats))<br><br>Filters: English language, human studies, articles, and reviews.

Figure S1. PRISMA flow diagram

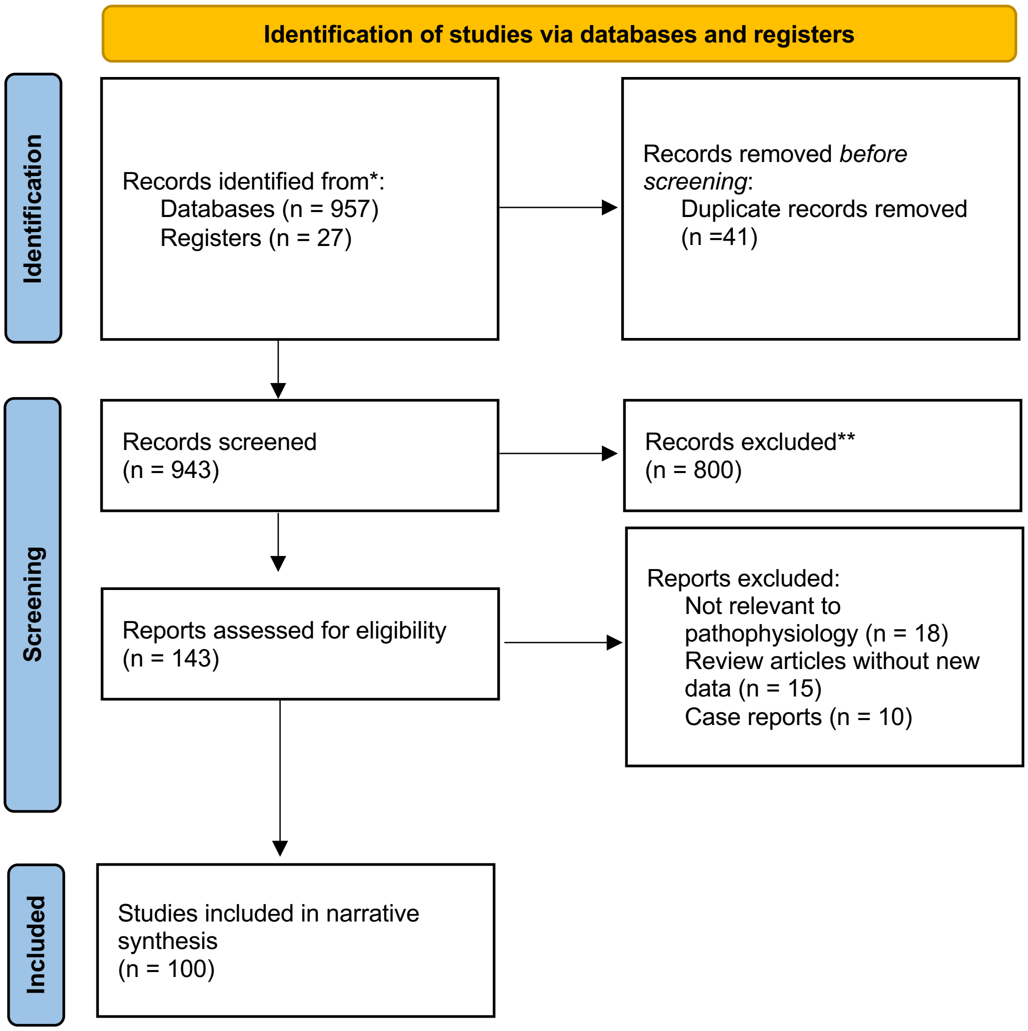

*Consider, if feasible to do so, reporting the number of records identified from each database or register searched (rather than the total number across all databases/registers).

**If automation tools were used, indicate how many records were excluded by a human and how many were excluded by automation tools.

**Table S2.** Summary of included studies				
	Title	Authors	Year	Journal
1	Abnormalities of placenta implantation	da Cunha Castro EC, Popek E	2018	Apmis
2	Human placenta and trophoblast development: key molecular mechanisms and model systems	Knöfler M, Haider S, Saleh L, Pollheimer J, Gamage T, James J	2019	Cell Mol Life Sci
3	Modeling Trophoblast Cell-Guided Uterine Spiral Artery Transformation in the Rat	Shukla V, Soares MJ	2022	Int J Mol Sci
4	The etiology of preeclampsia	Jung E, Romero R, Yeo L, Gomez-Lopez N, Chaemsaithong P, Jaovisidha A, et al.	2022	Am J Obstet Gynecol
5	Immune and Apoptosis Mechanisms Regulating Placental Development and Vascularization in Preeclampsia	Raguema N, Moustadraf S, Bertagnolli M	2020	Front Physiol
6	Role of the Monocyte-Macrophage System in Normal Pregnancy and Preeclampsia	Vishnyakova P, Elchaninov A, Fatkhudinov T, Sukhikh G	2019	Int J Mol Sci
7	Uterine NK cells and macrophages in pregnancy	Faas MM, de Vos P	2017	Placenta
8	The T helper type 17/regulatory T cell paradigm in pregnancy	Figueiredo AS, Schumacher A	2016	Immunology
9	Cell death mechanisms and their roles in pregnancy related disorders	Kasture V, Sahay A, Joshi S	2021	Adv Protein Chem Struct Biol
10	An integral role of mitochondrial function in the pathophysiology of preeclampsia	Kobayashi H, Yoshimoto C, Matsubara S, Shigetomi H, Imanaka S	2024	Mol Biol Rep
11	Establishment of trimester-specific reference intervals of serum lipids and the associations with pregnancy complications and adverse perinatal outcomes: a population-based prospective study	Lu Y, Jia Z, Su S, Han L, Meng L, Tang G, et al.	2021	Ann Med
12	Placental secretion of apolipoprotein A1 and E: the anti-atherogenic impact of the placenta	Melhem H, Kallol S, Huang X, Lüthi M, Ontsouka CE, Keogh A, et al.	2019	Sci Rep
13	Is maternal lipid profile in early pregnancy associated with pregnancy complications and blood pressure in pregnancy and long term postpartum?	Adank MC, Benschop L, Peterbroers KR, Smak Gregoor AM, Kors AW, Mulder MT, et al.	2019	Am J Obstet Gynecol
14	Foetoplacental communication via extracellular vesicles in normal pregnancy and preeclampsia	Chiarello DI, Salsoso R, Toledo F, Mate	2018	Mol Aspects Med

		A, Vázquez CM, Sobrevia L		
15	Update of syncytiotrophoblast derived extracellular vesicles in normal pregnancy and preeclampsia	Tannetta D, Masliukaite I, Vatish M, Redman C, Sargent I	2017	J Reprod Immunol
16	A proteomic analysis of placental trophoblastic cells in preeclampsia-eclampsia	Ma K, Jin H, Hu R, Xiong Y, Zhou S, Ting P, et al.	2014	Cell Biochem Biophys
17	Placental Vesicles Carry Active Endothelial Nitric Oxide Synthase and Their Activity is Reduced in Preeclampsia	Motta-Mejia C, Kandzija N, Zhang W, Mhlomi V, Cerdeira AS, Burdujan A, et al.	2017	Hypertension
18	Detrimental arterial inflammatory effect of microparticles circulating in preeclamptic women: ex vivo evaluation in human arteries	Boisramé-Helms J, Meziani F, Sananès N, Boisramé T, Langer B, Schneider F, et al.	2015	Fundam Clin Pharmacol
19	Oxidative Stress and Preeclampsia-Associated Prothrombotic State	Han C, Huang P, Lyu M, Dong J	2020	Antioxidants (Basel)
20	Markers of Endothelial Dysfunction Are Attenuated by Resveratrol in Preeclampsia	Bueno-Pereira TO, Bertozzi-Matheus M, Zampieri GM, Abbade JF, Cavalli RC, Nunes PR, et al.	2022	Antioxidants (Basel)
21	Imbalances in circulating angiogenic factors in the pathophysiology of preeclampsia and related disorders	Rana S, Burke SD, Karumanchi SA	2022	Am J Obstet Gynecol
22	Endothelial dysfunction and preeclampsia: role of oxidative stress	Sánchez-Aranguren LC, Prada CE, Riaño-Medina CE, Lopez M	2014	Front Physiol
23	Placental expression of eNOS, iNOS and the major protein components of caveolae in women with pre-eclampsia	Smith-Jackson K, Hentschke MR, Poli-de-Figueiredo CE, Pinheiro da Costa BE, Kurlak LO, Broughton Pipkin F, et al.	2015	Placenta
24	Resveratrol and grape juice: Effects on redox status and nitric oxide production of endothelial cells in in vitro preeclampsia model	Caldeira-Dias M, Viana-Mattioli S, de Souza Rangel Machado J, Carlström M, de Carvalho	2021	Pregnancy Hypertens

		Cavalli R, Sandrim VC		
25	Understanding the Role of Chemerin in the Pathophysiology of Pre-Eclampsia	Pankiewicz K, Issat T	2023	Antioxidants (Basel)
26	Evaluation of oxidative stress markers in subtypes of preeclampsia: A systematic review and meta-analysis	Freire VAF, Melo AD, Santos HL, Barros-Pinheiro M	2023	Placenta
27	Syncytiotrophoblast stress in preeclampsia: the convergence point for multiple pathways	Redman CWG, Staff AC, Roberts JM	2022	Am J Obstet Gynecol
28	Cross-Talk between Oxidative Stress and Inflammation in Preeclampsia	Tenório MB, Ferreira RC, Moura FA, Bueno NB, de Oliveira ACM, Goulart MOF	2019	Oxid Med Cell Longev
29	Markers of Endothelial Injury and Dysfunction in Early- and Late-Onset Preeclampsia	Kornacki J, Wirstlein P, Wender-Ozegowska E	2020	Life (Basel)
30	Oxidative stress in placental pathology	Schoots MH, Gordijn SJ, Scherjon SA, van Goor H, Hillebrands JL	2018	Placenta
31	Role of leptin in the pathophysiology of preeclampsia	Zeng S, Liu Y, Fan P, Yang L, Liu X	2023	Placenta
32	Chemerin: A Functional Adipokine in Reproductive Health and Diseases	Yu M, Yang Y, Huang C, Ge L, Xue L, Xiao Z, et al.	2022	Biomedicines
33	Chemerin regulates normal angiogenesis and hypoxia-driven neovascularization	Ben Dhaou C, Mandi K, Frye M, Acheampong A, Radi A, De Becker B, et al.	2022	Angiogenesis
34	Is Mitochondrial Oxidative Stress a Viable Therapeutic Target in Preeclampsia?	Vaka R, Deer E, LaMarca B	2022	Antioxidants (Basel)
35	Therapeutically targeting mitochondrial redox signalling alleviates endothelial dysfunction in preeclampsia	McCarthy C, Kenny LC	2016	Sci Rep
36	Mitochondrial Dysfunction in the Pathogenesis of Preeclampsia	Hu XQ, Zhang L	2022	Curr Hypertens Rep
37	Mitochondrial reactive oxygen species (ROS) and ROS-induced ROS release	Zorov DB, Juhaszova M, Sollott SJ	2014	Here is the table based on the 77 articles provided for analysis, split into three parts:
38	Diphenyl diselenide regulates Nrf2/Keap-1 signaling pathway and counteracts hepatic oxidative stress induced by bisphenol A in male mice	Müller SG, Jardim NS, Quines CB, Nogueira CW	2018	Environ Res

39	Oxidative stress activated by Keap-1/Nrf2 signaling pathway in pathogenesis of preeclampsia	Feng H, Wang L, Zhang G, Zhang Z, Guo W	2020	Int J Clin Exp Pathol
40	Bioactive factors in uteroplacental and systemic circulation link placental ischemia to generalized vascular dysfunction in hypertensive pregnancy and preeclampsia	Shah DA, Khalil RA	2015	Biochem Pharmacol
41	The Impact of Iron Overload and Ferroptosis on Reproductive Disorders in Humans: Implications for Preeclampsia	Ng SW, Norwitz SG, Norwitz ER	2019	Int J Mol Sci
42	Hypoxia and Placental Development	Soares MJ, Iqbal K, Kozai K	2017	Birth Defects Res
43	Oxidative Stress in Preeclampsia and Placental Diseases	Aouache R, Biquard L, Vaiman D, Miralles F	2018	Int J Mol Sci
44	Evaluation of Glutathione Peroxidase 4 role in Preeclampsia	Peng X, Lin Y, Li J, Liu M, Wang J, Li X, et al.	2016	Sci Rep
45	Ferroptosis and Its Emerging Role in Pre-Eclampsia	Chen Z, Gan J, Zhang M, Du Y, Zhao H	2022	Antioxidants (Basel)
46	Oxidized arachidonic and adrenic PEs navigate cells to ferroptosis	Kagan VE, Mao G, Qu F, Angeli JP, Doll S, Croix CS, et al.	2017	Nat Chem Biol
47	Lipid peroxidation in cell death	Gaschler MM, Stockwell BR	2017	Biochem Biophys Res Commun
48	Preeclampsia: From Inflammation to Immunoregulation	Cornelius DC	2018	Clin Med Insights Blood Disord
49	Association between cytokine profile and transcription factors produced by T-cell subsets in early- and late-onset pre-eclampsia	Ribeiro VR, Romao-Veiga M, Romagnoli GG, Matias ML, Nunes PR, Borges VTM, et al.	2017	Immunology
50	Regulatory T-cells and preeclampsia: an overview of literature	Rahimzadeh M, Norouzian M, Arabpour F, Naderi N	2016	Expert Rev Clin Immunol
51	Regulatory T-cell Subpopulations in Severe or Early-onset Preeclampsia	Boij R, Mjösberg J, Svensson-Arvelund J, Hjorth M, Berg G, Matthiesen L, et al.	2015	Am J Reprod Immunol
52	Pregnancy-associated diseases are characterized by the composition of the systemic regulatory T cell (Treg) pool with distinct subsets of Tregs	Steinborn A, Schmitt E, Kisielewicz A, Rechenberg S, Seissler N, Mahnke K, et al.	2012	Clin Exp Immunol

53	Gene Polymorphism in Five Target Genes of Immunosuppressive Therapy and Risk of Development of Preeclampsia	Previtera F, Restaino S, Romano G, Vizzielli G, Neri A, Scalzotto E, et al.	2021	Healthcare (Basel)
54	Genetic variants in 3'-UTRs of MTHFR in the pregnancies complicated with preeclampsia and bioinformatics analysis	Mohammadpour-Gharehbagh A, Salimi S, Keshavarzi F, Saeidian F, Mousavi M, Teimoori B, et al.	2018	J Cell Biochem
55	Association of MicroRNA-155rs767649 Polymorphism with Susceptibility to Preeclampsia	Ayoub SE, Shaker OG, Abdelwahed MY, Ahmed NA, Abdelhameed HG, Bosilah AH, et al.	2019	Int J Mol Cell Med
56	MicroRNAs: New Players in the Pathobiology of Preeclampsia	Bounds KR, Chiasson VL, Pan LJ, Gupta S, Chatterjee P	2017	Front Cardiovasc Med
57	Analysis of Complement C3 Gene Reveals Susceptibility to Severe Preeclampsia	Lokki AI, Kaartokallio T, Holmberg V, Onkamo P, Koskinen LLE, Saavalainen P, et al.	2017	Front Immunol
58	Polymorphisms within the Tumor Necrosis Factor-Alpha Gene Is Associated with Preeclampsia in Taiwanese Han Populations	Lin CW, Chen CH, Wu MH, Chang FM, Kang L	2023	Biomedicines
59	Preeclampsia: Updates in Pathogenesis, Definitions, and Guidelines	Phipps E, Prasanna D, Brima W, Jim B	2016	Clin J Am Soc Nephrol
60	Functional polymorphisms of NOS3 and GUCY1A3 affect both nitric oxide formation and association with hypertensive disorders of pregnancy	Pereira DA, Luizon MR, Palei AC, Tanus-Santos JE, Cavalli RC, Sandrim VC	2024	Front Genet
61	Mitochondria and Coenzyme Q10 in the Pathogenesis of Preeclampsia	Teran E, Hernández I, Tana L, Teran S, Galaviz-Hernandez C, Sosa-Macías M, et al.	2018	Front Physiol
62	CoQ10 alleviate preeclampsia symptoms by enhancing the function of mitochondria in the placenta of pregnant rats with preeclampsia	Xu X, Pan JR, Zhang YZ	2019	Hypertens Pregnancy

63	Preeclampsia: Platelet procoagulant membrane dynamics and critical biomarkers	Agbani EO, Skeith L, Lee A	2023	Res Pract Thromb Haemost
64	Placental thromboinflammation impairs embryonic survival by reducing placental thrombomodulin expression	Kohli S, Singh KK, Gupta A, Markmeyer P, Lochmann F, Gupta D, et al.	2021	Blood
65	The Role of the Renin-Angiotensin-Aldosterone System in Preeclampsia: a Review	Gathiram P, Moodley J	2020	Curr Hypertens Rep
66	Association between micronutrients, oxidative stress biomarkers and angiogenic growth mediators in early and late-onset preeclamptic Ghanaian women	Anto EO, Ofori Boadu WI, Addai-Mensah O, Wiafe YA, Owiredu WK, Obirikorang C, et al.	2023	SAGE Open Med
67	Placental lesions and differential expression of pro- and anti-angiogenic growth mediators and oxidative DNA damage marker in placentae of Ghanaian suboptimal and optimal health status pregnant women who later developed preeclampsia	Anto EO, Coall DA, Asiamah EA, Afriyie OO, Addai-Mensah O, Wiafe YA, et al.	2022	PLoS One
68	The Role of Oxidative Stress, Adhesion Molecules and Antioxidants in Preeclampsia	Haram K, Mortensen JH, Myking O, Magann EF, Morrison JC	2019	Curr Hypertens Rev
69	Association of oxidative DNA damage, protein oxidation and antioxidant function with oxidative stress induced cellular injury in pre-eclamptic/eclamptic mothers during fetal circulation	Negi R, Pande D, Karki K, Kumar A, Khanna RS, Khanna HD	2014	Chem Biol Interact
70	Putative Key Role of Inositol Messengers in Endothelial Cells in Preeclampsia	Kunjara S, McLean P, Rademacher L, Rademacher TW, Fascilla F, Bettocchi S, et al.	2016	Int J Endocrinol
71	Placental Genetic Variants in the Upstream Region of the FLT1 Gene in Pre-eclampsia	Ohwaki A, Nishizawa H, Kato A, Kato T, Miyazaki J, Yoshizawa H, et al.	2020	J Reprod Infertil
72	Molecular mechanisms and therapeutic implications of the carbon monoxide/hmox1 and the hydrogen sulfide/CSE pathways in the prevention of pre-eclampsia and fetal growth restriction	Ahmed A	2014	Pregnancy Hypertens
73	Modulation of EndMT by Hydrogen Sulfide in the Prevention of Cardiovascular Fibrosis	Testai L, Brancaleone V, Flori L, Montanaro R, Calderone V	2021	Antioxidants (Basel)

74	Interaction Between NOS3 and HMOX1 on Antihypertensive Drug Responsiveness in Preeclampsia	Sandrim VC, Luizon MR, Pilan E, Caldeira-Dias M, Coeli-Lacchini FB, Kors G, et al.	2020	Rev Bras Ginecol Obstet
75	Downregulation of LncRNA-MEG3 promotes HTR8/SVneo cells apoptosis and attenuates its migration by repressing Notch1 signal in preeclampsia	Wang R, Zou L	2020	Reproduction
76	Down-regulated long non-coding RNA MEG3 and its effect on promoting apoptosis and suppressing migration of trophoblast cells	Zhang Y, Zou Y, Wang W, Zuo Q, Jiang Z, Sun M, et al.	2015	J Cell Biochem
77	Long noncoding RNA maternally expressed gene 3 improves trophoblast dysfunction and inflammation in preeclampsia through the Wnt/β-Catenin/nod-like receptor pyrin domain-containing 3 axis	Liang Y, Wang P, Shi Y, Cui B, Meng J	2022	Front Mol Biosci
78	Placental glycosylation senses the anti-angiogenic milieu induced by human sFLT1 during pregnancy	K. Kirkgöz, R. Vogtmann, Y. Xie, F. Zhao, A. Riedel, L. M. Adam, et al.	2024	J Reprod Immunol
79	Maternal-derived galectin-1 shapes the placenta niche through Sda terminal glycosylation: Implication for preeclampsia	Y. Xie, F. Zhao, N. Freitag, S. Borowski, Y. Wang, C. Harms, et al.	2023	PNAS Nexus
80	Pregnancy Galectinology: Insights Into a Complex Network of Glycan Binding Proteins	S. M. Blois, G. Dveksler, G. R. Vasta, N. Freitag, V. Blanchard and G. Barrientos	2019	Front Immunol
81	Oxidative stress in pregnancy complicated by preeclampsia	S. San Juan-Reyes, L. M. Gómez-Oliván, H. Islas-Flores and O. Dublán-García	2020	Arch Biochem Biophys
82	Serum uric acid to creatinine ratio and risk of preeclampsia and adverse pregnancy outcomes	F. Piani, D. Agnoletti, A. Baracchi, S. Scarduelli, C. Verde, G. Tossetta, et al.	2023	J Hypertens
83	First Trimester CD93 as a Novel Marker of Preeclampsia and Its Complications: A Pilot Study	F. Piani, G. Tossetta, S. Fantone, C. Agostinis, N. Di Simone, M. Mandalà, et al.	2023	High Blood Press Cardiovasc Prev
84	Modulation of NRF2/KEAP1 Signaling in Preeclampsia	G. Tossetta, S. Fantone, F. Piani, C. Crescimanno, A.	2023	Cells

		Ciavattini, S. R. Giannubilo, et al.		
85	Learning from the placenta: acute atherosis and vascular remodeling in preeclampsia-novel aspects for atherosclerosis and future cardiovascular health	A. C. Staff, R. Dechend and R. Pijnenborg	2010	Hypertension
86	Preeclampsia and uteroplacental acute atherosis: immune and inflammatory factors	A. C. Staff, G. M. Johnsen, R. Dechend and C. W. G. Redman	2014	J Reprod Immunol
87	Clinical importance of the human umbilical artery potassium channels	M. Lorigo, N. Oliveira and E. Cairrao	2020	Cells
88	Susceptibility of male reproductive system to bisphenol A, an endocrine disruptor: Updates from epidemiological and experimental evidence	S. K. Yadav, V. Bijalwan, S. Yadav, K. Sarkar, S. Das and D. P. Singh	2023	J Biochem Mol Toxicol
89	Exposure to endocrine-disrupting chemicals and risk of gestational hypertension and preeclampsia: A systematic review and meta-analysis	A. Hirke, B. Varghese, S. Varade and R. Adela	2023	Environ Pollut
90	Human exposure to bisphenol A (BPA) through medical-hospital devices: A systematic review	A. G. C. Guimarães, V. L. Coutinho, A. Meyer, P. C. Lisboa and E. G. de Moura	2023	Environ Toxicol Pharmacol
91	Plastics derived endocrine-disrupting compounds and their effects on early development	S. Basak, M. K. Das and A. K. Duttaroy	2020	Birth Defects Res
92	Association of pro- and anti-inflammatory cytokines in preeclampsia	R. Aggarwal, A. K. Jain, P. Mittal, M. Kohli, P. Jawanjal and G. Rath	2019	J Clin Lab Anal
93	In Vitro Effects of Bisphenol A β-D-Glucuronide (BPA-G) on Adipogenesis in Human and Murine Preadipocytes	J. G. Boucher, A. Boudreau, S. Ahmed and E. Atlas	2015	Environ Health Perspect
94	Bisphenol a and reproductive health: update of experimental and human evidence, 2007-2013	J. Peretz, L. Vrooman, W. A. Ricke, P. A. Hunt, S. Ehrlich, R. Hauser, et al.	2014	Environ Health Perspect
95	Personal care product use and urinary phthalate metabolite and paraben concentrations during pregnancy among women from a fertility clinic	J. M. Braun, A. C. Just, P. L. Williams, K. W. Smith, A. M. Calafat and R. Hauser	2014	J Expo Sci Environ Epidemiol
96	Plastics derived endocrine disruptors (BPA, DEHP and DBP) induce epigenetic transgenerational inheritance of obesity, reproductive disease and sperm epimutations	M. Manikkam, R. Tracey, C. Guerrero-	2013	PLoS One

		Bosagna and M. K. Skinner		
97	Relationship between Androgens and Vascular and Placental Function during Pre-eclampsia	L. M. Fernandes, M. Lorigo and E. Cairrao	2024	Curr Issues Mol Biol
98	Androgens in maternal vascular and placental function: implications for preeclampsia pathogenesis	S. Kumar, G. H. Gordon, D. H. Abbott and J. S. Mishra	2018	Reproduction
99	Maternal serum hormonal factors in the pathogenesis of preeclampsia	E. M. Salustiano, J. C. De Pinho, K. Provost, R. Ruano and M. Zugaib	2013	Obstet Gynecol Surv
100	Estrogen regulation of placental angiogenesis and fetal ovarian development during primate pregnancy	E. D. Albrecht and G. J. Pepe	2010	Int J Dev Biol
